# 

**Published:** 1894-01

**Authors:** 


### Society Meetings.

The eighty-eighth annual meeting of the Medical Society of the State of New York will be held in the City Hall, at Albany, Tuesday, Wednesday and Thursday, February 6, 7 and 8, 1894, under the presidency of Dr. Herman Bendell, of Albany. An elaborate program is preparing and a large attendance of members and guests is anticipated.

The Association of Erie Railway Surgeons will hold its next annual meeting on Friday, January 5, 1894, at the Academy of Medicine, 17 West Forty-third street, New York City. An interesting program has been published and an interesting meeting is anticipated. The officers for this meeting are: President, Dr. R.

Sayre Harnden, Waverly, N. Y.; Vice-President, Dr. C. B. Kibler, Corry, Pa.; Secretary and Treasurer, Dr. W. W. Appley, Cochecton, N. Y. Executive Committee: Dr. C. M. Daniels, Buffalo, N. Y.; Dr. J. L. Eddy, Olean, N. Y.; and Dr. C. B. Kibler, Corry, Pa.

---

The Medical Society of the County of Chautauqua held its semi-annual meeting at the Sherman House, in Jamestown, Tuesday, December 12, 1893. An interesting program was published and the subject of typhoid fever was discussed. The attendance was large and the discussions spirited and instructive. The President, Dr. Nelson G. Richmond, of Fredonia, is to be congratulated upon the successful inauguration of his administration and in rejuvenating this excellent society, that had of late grown somewhat apathetic with reference to medical affairs. In his good work he was ably seconded by Dr. George E. Blackham, of Dunkirk, who proved a most efficient lieutenant in every sense of the word.

---

Dr. A. Jacobi, chairman of the American National Committee of the Eleventh International Medical Congress, has received the following communication from the Secretary-General:

*First.* Papers to be read in any of the sections of the Congress should be announced on or before January 31, 1894, to the secretary-general, Prof. E. Maragliano, Ospedale Pammatone, Genova, Italy.

*Second.* The title of the paper ought to be accompanied with a brief abstract of its contents and conclusions.

*Third.* The program to be distributed will contain the titles of all the papers announced before August 31, 1893, and since.

*Fourth.* The reductions granted by the railway companies months ago will be available from March 1st to April 30, 1894.

In the interest of such medical men as will sail for Europe before official cards will have been received from the general committee, Dr. Jacobi proposes to supply, in as official a form as he thinks he is justified in doing, credentials which are expected to be of some practical value. It is suggested, besides, that a passport may increase the traveler's facilities.

A letter of the secretary-general's, dated November 29th, states that traveling documents will be sent to the address of every subscriber on or before February 15, 1894; and that after that date congressists will have to apply to Dr. Jacobi.

It also contains the following regulations of former circulars: Members' dues are five dollars, (money order to Prof. L. Pagliani, Rome,) guests' (wives and adult relations), two dollars; medical students, no fees. All are entitled to traveling documents.

Reductions on the Italian railways are available from March 1st until April 30th.

### BOOKS RECEIVED.

Foreign Bodies in the Larynx and Trachea and in the Pharynx and Esophagus. By John O. Roe, M. D., Rochester, N. Y., Fellow of the American Laryngological Association; Corresponding Member of the Société Francaise d'Otologie, de Laryngologie et de Rhinologie; Member of the British Medical Association, of the American Climatological Association, of the American Medical Association, of the Medical Society of the State of New York, of the Central New York Medical Association, of the Monroe County Medical Society, etc. Large octavo, pp. 73. Reprinted from Volume II. of The System of Diseases of the Ear, Nose, and Throat. Edited by Charles H. Burnett, M. D. Published by J. B. Lippincott Company, Philadelphia. 1893.

Annual Report of the State Board of Charities for the year 1892. Transmitted to the Legislature January 26, 1893. Octavo, pp. 591. Albany: James B. Lyons, State Printer. 1893.

New Truths in Ophthalmology as developed by G. C. Savage, M. D., Professor of Ophthalmology in the Medical Departments of the University of Nashville and Vanderbilt University. Thirty-two illustrations, 12mo, pp. 160. Published by the author. Printed at the Publishing House of the M. E. Church, South Nashville, Tenn. 1893.

Transactions of the Medical and Chirurgical Faculty of the State of Maryland. Ninty-fourth annual session, held at Baltimore, Maryland, April, 1892; also semi-annual session, held at Rockville, Md., November, 1891. Octavo, pp. 124. Baltimore: Griffin, Curley & Co., Printers, 202 E. Baltimore street. 1892.

Statistics of Public Libraries in the United States and Canada. By Weston Flint, Statistician of the Bureau of Education, Bureau of Education Circular of Information, No. 7, 1893. Octavo, pp. 226. Washington: Government Printing Office. 1893.

Report of the Surgeon-General of the Army to the Secretary of War for the fiscal year ending June 30, 1893. Octavo, pp. 231. Washington: Government Printing Office. 1893.

New York County Medical Association, State of New York. Register of Members, Manual of Information. Duodecimo, pp. 116. New York: Published by the Association, 1893.

Transactions of the Medical and Chirurgical Faculty of the State of Maryland. Ninety-fifth annual session, held at Baltimore, Md., April, 1893; also semi-annual session, held at Easton, Md., November, 1892. Paper, octavo, pp. 111. Baltimore: Griffin, Curley & Co., Printers, 202 E. Baltimore street. 1893.

Syllabus of Lectures on the Practice of Surgery, arranged in conformity with the American Text-book of Surgery. By N. Senn, M. D., Ph. D., LL. D., Chicago; Professor of the Practice of Surgery and Clinical Surgery in Rush Medical College; Professor of Surgery in the Chicago Polyclinic; Attending Surgeon to Presbyterian Hospital; Surgeon-in-Chief St. Joseph's Hospital, etc., etc. Philadelphia: W. B. Saunders, 925 Walnut street. 1894.

---

### Academy of Medicine Notes.

### SECTION MEETINGS FOR JANUARY.

TUESDAY evening, January 2, 1894, Section on Surgery. The programme for this meeting is as follows: Symptomatology and Causes of Urethral Stricture, Dr. William H. Bergtold; Treatment of Urethral Stricture, Dr. William C. Phelps.

Tuesday evening, January 9th, Section on Medicine.

Thursday evening, January 16th, Section on Anatomy, Physiology and Pathology.

Tuesday evening, January 23d, Section on Obstetrics and Gynecology.

THE Columbian Calendar, for 1894, for the use of physicians and business men, is already at hand. It is a pad calendar to stand upon the desk, and is of convenient shape and size, with a separate leaf for each day, which contains something interesting for every bicyclist. This calendar for 1894 is especially attractive.

JOURNALISTIC CHANGES.— The *Pacific Medical Record* has changed its name to *The Medical Sentinel.* It will be continued to be published at Portland, Oregon, under the same editorial and business management as heretofore.

The Fort Wayne *Medical Magazine* made its first appearance in December as a continuation of McCaskey's Clinical Studies. It has enlarged its editorial staff, increased its size, and presents a very handsome appearance.

MELVIL DEWEY, Secretary of the Board of Regents, has prepared a capital little handbook, giving in brief compass a clear outline of the work of the time-honored University of the State of New York. The object, government, powers and duties and meetings of the Board of Regents are succinctly described; the operation of the administrative departments—executive office, examinations, extension, State Library and State Musem—is explained; and a considerable mass of interesting information is condensed into a few pages. It is an illustrated pamphlet, small enough to be slipped into an ordinary letter envelope, and can be had free on application at the State Library, or office of the Board of Regents.—*New York Tribune.*

---

MR. W. B. SAUNDERS, medical publisher, of Philadelphia, sends out to the profession the following circular:

DEAR DOCTOR: I take pleasure in sending you herewith press opinions of Dr. Pepper's first volume of Theory and Practice of Medicine. We now have the entire manuscript of the second volume in our printer's hands, and we can assure the profession at large that the work will be placed in their hands within the next six weeks. There is one feature about this book to which I would call your special attention. In the first volume nearly 200 pages are from the pen of Dr. William Pepper, and in the second volume he writes over 300 pages, thus making over one-fourth of the entire work from the editor.

I now have in press ready for early publication, American Text-Book of Gynecology, an announcement of which I enclose you. You will see that this work makes a special feature in its illustrations. I will also have ready within the next two months American Text-Book of Diseases of Children, which will be edited by Dr. Louis Starr. This work, as well as the Gynecology, will contain a large number of beautiful illustrations, a great number of which are originals. In point of perfection, it will be far in advance of any single volume work on children yet issued, and will contain many colored and full-page illustrations, as is usually put in works of four and five volumes; yet, the price will be uniform with my American Text-Book of Surgery.

We will send you by mail in a few days a copy of Senn's Syllabus of the American Text-Book of Surgery, which we think will be a valuable aid to all who now possess the American Surgery. This latter book, as I have written you before, has had a phenomenal sale, over 10,000 copies having been sold last year. By the present indications, I am sure my American Text-Book of Gynecology, and also the one on Diseases of Children, will meet with as flattering a reception as the Surgery.

Musical Contest.—We have received from the publishers the two great rival marches, "Protective Tariff Grand March" and "Free Trade Grand March." The former is by the well-known author, Will L. Thompson, of East Liverpool, Ohio. The latter is by Wm. Lamartine, an author of equal talent, and both pieces are beautiful, bright and showy marches of medium difficulty for the piano or organ. Price, forty cents each. They are for sale at all music stores, or may be procured from Mr. Thompson at one-half price. One firm alone has ordered 15,000 copies.

---

The second edition of the December World's Fair *Cosmopolitan* brings the total up to the extraordinary figure of 400,000 copies, an unprecedented result in the history of magazines. Four hundred thousand copies—200 tons—94,000,000 pages—enough to fill 200 wagons with 2,000 pounds each; in a single line, in close order, this would be a file of wagons more than a mile and a half long. This means not less than 2,000,000 readers, scattered throughout every town and village in the United States. The course of *The Cosmopolitan* for the past twelve months may be compared to that of a rolling snowball; more subscribers mean more money spent in buying the best articles and best illustrations in the world; better illustrations and better articles mean more subscribers, and so the two things are acting and reacting upon each other, until it seems probable that the day is not far distant when the magazine publisher will be able to give so excellent an article that it will claim the attention of every intelligent reader in the country.

---

E. B. Treat, publisher, New York, has in press for early publication the 1894 *International Medical Annual*, being the twelfth yearly issue of this eminently useful work. Since the first issue of this one volume reference work, each year has witnessed marked improvements; and the prospectus of the forthcoming volume gives promise that it will surpass any of its predecessors. It will be the conjoint authorship of forty-one distinguished specialists, selected from the most eminent physicians and surgeons of America, England, and the Continent. It will contain complete reports of the progress of medical science in all parts of the world, together with a large number of original articles and reviews on subjects with which the authors' names are especially associated. In short, the design of the book is, while not neglecting the specialist, to bring the general practitioner into direct communication with those who are advancing the science of medicine, so he may be furnished with all that is worthy of preservation, as reliable aids in his daily work. Illustrations in black and colors will be consistently used wherever helpful in elucidating the text. Altogether, it makes a most useful, if not absolutely indispensable, investment for the medical practitioner. While the book will be so much improved over previous issues, the price will remain the same as heretofore, $2.75.

Mr. Treat also has in press the following books, which will be issued early in 1894:

A Manual of Clinical Diagnosis, by Albert Abrams, M. D., assistant professor of clinical medicine and demonstrator of pathology, Cooper Medical College, San Francisco, new and enlarged edition, $2.75.

Diseases of the Hair and Scalp, by Gen. T. Jackson, M. D., chief of dermatological clinic, college of Physicians and Surgeons, New York; illustrated, second edition, enlarged. $2.75.

How to Use the Forceps: a manual of the obstetric art and mechanism of labor, by Prof. H. G. Landis, M. D., Columbus, O.; enlarged edition, by C. M. Bushong, M. D., New York, $1.75.

Also just issued: Practical Hygiene, based on modern theories and scientific progress, by C. G. Currier, M. D., New York, specialist and expert in sanitary science, $2.75.

Mathews' Medical Quarterly, to be devoted to diseases of the rectum, and gastro-intestinal diseases, rectal and gastro-intestinal surgery, is announced to be issued early in January, 1894. The editor, Dr. Joseph M. Mathews, is the well-known author on the subjects to which this magazine will be devoted. It will be published in Louisville, Ky., and Dr. Henry E. Luley is the assistant editor and manager.

---

THE SAMUEL D. GROSS PRIZE.—The Quinquennial prize of $1000, under the will of the late Samuel D. Gross, M.D., will be awarded January 1, 1895. The conditions annexed by the testator are that the prize "shall be awarded every five years to the writer of the best original essay, not exceeding 150 printed pages, octavo, in length, illustrative of some subject in surgical pathology or surgical practice, founded upon original investigations, the candidates for the prize to be American citizens."

It is expressly stipulated that the successful competitor who receives the prize shall publish his essay in book form, and that he shall deposit one copy of the work in the Samuel D. Gross library of the Philadelphia Academy of Surgery.

The essays, which must be written by a single author, in the English language, should be sent to Dr. J. Ewing Mears, 1429 Walnut St., Philadelphia, before January 1, 1895.

Each essay must be distinguished by a motto, and accompanied by a sealed envelope bearing the same motto, and containing the name and address of the writer. No envelope will be opened except that which accompanies the successful essay.

The committee will return the unsuccessful essays if reclaimed by their respective writers, or their agents, within one year.

The committee reserves the right to make no award if the essays submitted are not considered worthy of the prize.

---

A Novel Literary Enterprise.—The American Coöperative Library, recently organized in New York, undertakes to give to book readers, anywhere in the United States, better facilities than heretofore given them by the largest libraries in the leading cities, and at an almost trifling cost. You order any book you want, suitable for general circulation, and it is supplied immediately; you can order either direct or through your local bookseller, country postmaster, or others acting as local agents. One cent a day for a dollar book, proportionately for other values, is the general basis of loans, three cents being the least charge made. Thus, "Ben Hur" costs four cents for three days, "The Prince of India" five cents for four days for each volume, "Lorna Doone" three cents for six days, "Uncle Tom's Cabin" three cents for eight days, and so on. You deposit the price of the book when you order it, keep it as long as you please, and on its return get any other book you want to borrow or want to buy. There are some special advantages to book clubs. Thus, at a cost of from $2.00 to $5.00 a year one can have access to the whole world of current and standard literature. Does not this bring the Literary Millennium pretty near every home? Circulars are sent free on request, or a 160-page catalogue for two cents. Address John B. Alden, Manager, 57 Rose street, New York.

---

Notice to Contributors.—We are glad to receive contributions from every one who knows anything of interest to the profession. Articles designed for publication in the Journal should be handed in before the first day of the month. The Editors are not responsible for the views or opinions of contributors. All communications should be addressed to the Managing Editor, 284 Franklin St., Buffalo, N. Y.

